# Torsion of Ovarian Dysgerminoma in a Child: Role of Computed Tomography

**DOI:** 10.7759/cureus.2522

**Published:** 2018-04-23

**Authors:** Kumail Khandwala, Jehanzeb Shahid, Naila Nadeem, Muhammad Usman U Tariq

**Affiliations:** 1 Department of Radiology, The Aga Khan University, Karachi, PAK; 2 Department of Pathology & Laboratory Medicine, The Aga Khan University, Karachi, PAK

**Keywords:** ovarian mass, germ cell tumor, torsion, dysgerminoma, pediatric, ct

## Abstract

Dysgerminomas are malignant germ cell tumors of the ovary that most commonly occur in the adolescent population. Ovarian dysgerminoma presenting with complications like torsion is a rare entity in the pediatric age group. Cross-sectional imaging plays a crucial role in diagnosis, tumor staging before surgical resection, and for planning adjuvant chemotherapy. We report a case of a nine-year-old female who presented to the emergency room (ER) with abdominal distention and abdominal pain. Computed tomography scan revealed a large right-sided pelvic mass with areas of low attenuation, speckled calcification, peritumoral free fluid, and a twisted vascular pedicle that was likely originating from the left adnexa. The right ovary was normal in appearance. Suspicion of a left-sided ovarian tumor with torsion was raised, which was later confirmed on surgery and histopathology of the resected specimen.

## Introduction

Ovarian germ cell tumors (GCTs) are derived from primordial germ cells of the ovary and can either be benign or malignant. Dysgerminomas are labeled as female counterparts of testicular seminomas and although accounting for only 1-2% of malignant ovarian neoplasms, they are the most commonly occurring malignant GCT in females less than 30 years with peak incidence between 15-19 years of age [1]. Histologically, they present as aggregates of large, uniformly appearing giant cells with no differentiation to embryonal or extraembryonal structures. Additionally, they are associated with elevated serum lactate dehydrogenase (LDH) and an additional elevated beta human chorionic gonadotropin (hCG) level in 5% of the patients, secondary to infiltration by syncytiotrophoblasts [1, 2].

Dysgerminomas are more frequently detected in adolescent women, especially during pregnancy and can be bilateral in 15% of the cases [1, 2]. Unlike most other germ cell tumors, they tend to grow rapidly and are usually diagnosed early at initial presentation. Patients often present with abdominal pain and distension. Because of the rapidly growing nature of the tumor, there may be associated complications like rupture, hemoperitoneum or torsion, and patients can present to the emergency department with an acute abdomen [1]. We report a case of a female child with a large malignant ovarian dysgerminoma who presented with signs of torsion of the tumor. This case report demonstrates the importance of both the clinical and radiological findings of an unusual presentation of this ovarian malignancy in a child.

## Case presentation

A nine-year-old girl presented to the emergency department with abdominal pain and distention for the past one week, with sudden increase in intensity of pain for the last four hours. The patient had not yet reached the age of menarche. There was no associated nausea or vomiting and her bowel habits were not affected. Past medical, surgical, and family history was also insignificant. An abdominal examination revealed tenderness in the lower abdomen with a firm palpable mass occupying the right side of the abdomen. Her blood counts showed an elevated total leukocyte count of 13,000 cells/dL with neutrophilic predominance. Initial clinical assessment raised the possibility of an appendicular mass.

**Figure 1:**
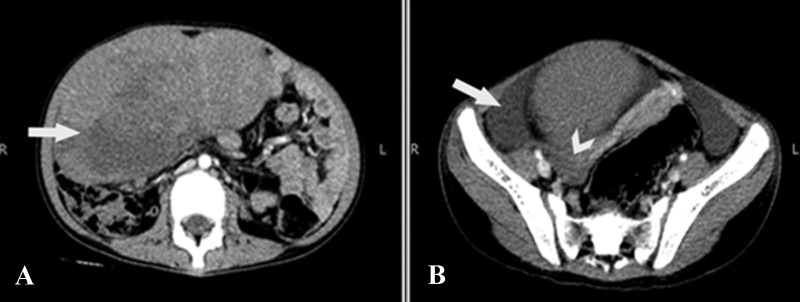
Computed tomography of the abdomen and pelvis axial sections A) Large right-sided lobulated pelvic mass with central areas of low attenuation suggestive of necrosis (arrow). B) There was free fluid adjacent to the lesion (arrow). The right ovary was separately visualised and appears normal (arrowhead).

The patient therefore immediately underwent a contrast-enhanced computed tomography (CT) scan of the abdomen and pelvis, which revealed a large soft tissue mass measuring approximately 80 x 150 x 170 mm in anteroposterior, transverse, and craniocaudal dimensions, respectively, and was predominantly occupying the right mid and lower quadrant. The mass showed some areas of low attenuation, suggestive of necrosis/intratumoral edema (Figure 1A). There was free fluid noted adjacent to the lesion and in the pelvis (Figure 1B). The right ovary was separately identified and appeared normal (Figure 1B).

**Figure 2:**
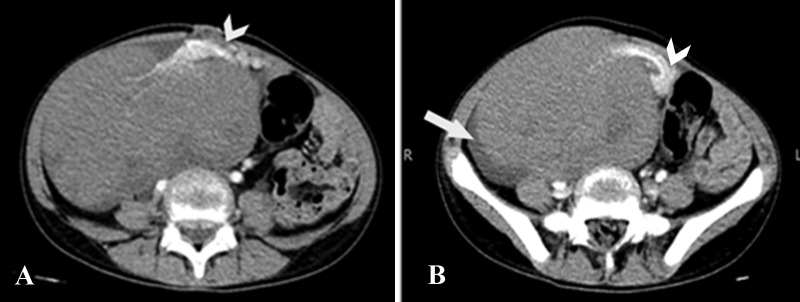
Computed tomography of the abdomen and pelvis axial sections A & B: Twisted vascular pedicle in the medial aspect of the mass, which was originating from the left adnexa (arrowheads). Free fluid seen adjacent to the lesion (arrow).

Anteromedially, the mass had a tortuous, twisted vascular pedicle that was likely originating from the left adnexa (Figure 2).

**Figure 3:**
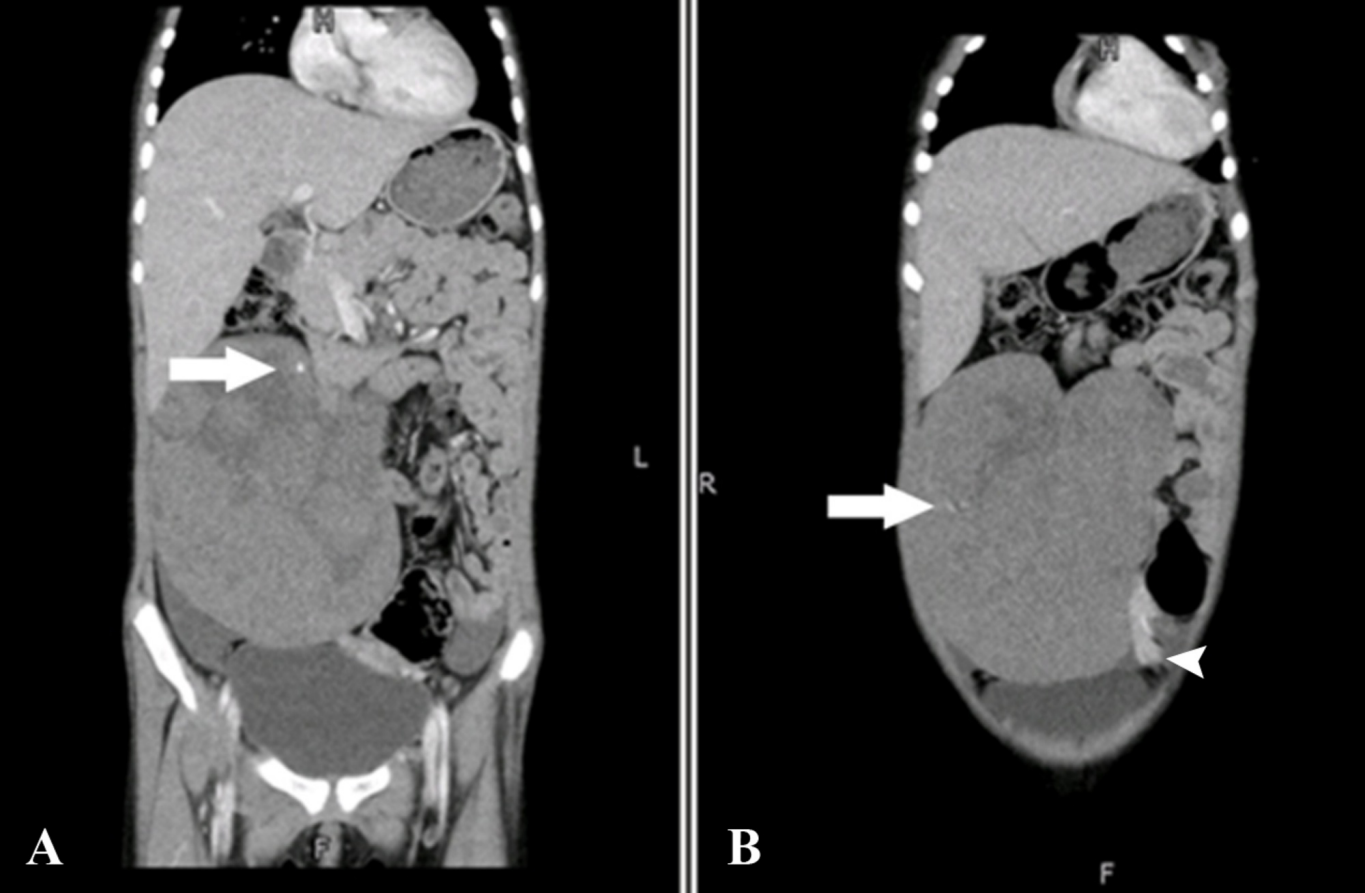
Computed tomography of the abdomen and pelvis coronal sections Speckled calcifications were noted in the mass (arrows). Partially visualised twisted vascular pedicle also seen (arrowhead).

Additionally, few speckled calcifications were noted in the mass (Figure 3). No enhancing fibrovascular septa were noted in the lesion.

No evidence of regional lymphadenopathy or distant metastases was found on the CT examination. On the basis of the radiological picture, an impression of left ovarian tumor with torsion was suggested.

The patient then underwent an exploratory laparotomy and left salpingo-oophorectomy along with partial omentectomy. Intraoperative findings included a large bilobed edematous mass weighing approximately 1.5 kg with a twisted, thickened vascular pedicle and varicosed vessels. The left fallopian tube was slightly thickened as well. No lymphadenopathy or invasion into the surrounding structures was seen.

**Figure 4:**
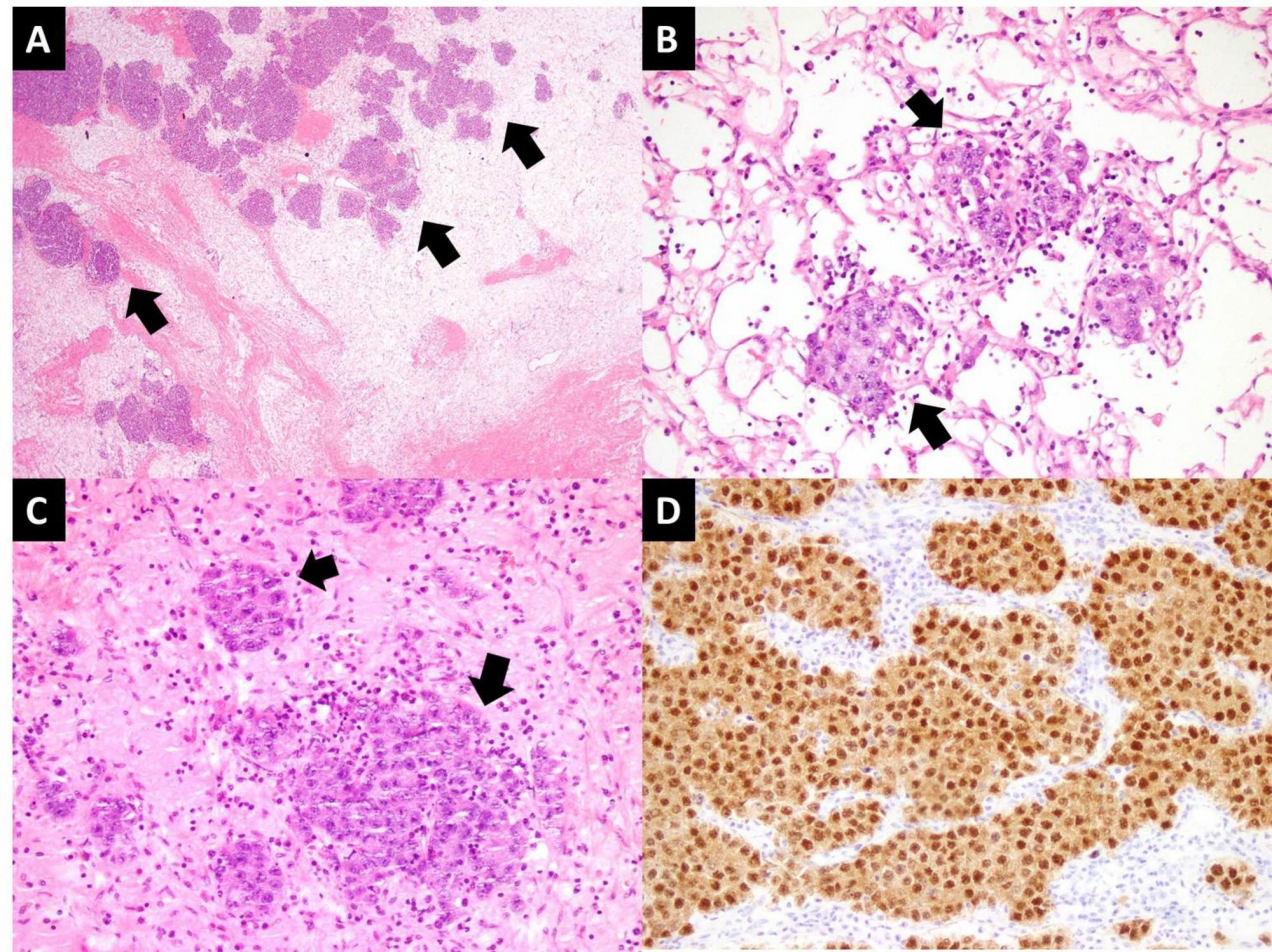
Histology slides A) Low power view of tumor showing edematous stroma and scattered tumor nests (arrows). B & C) Tumor nests (arrows) with surrounding dilated and congested thin walled vessels. D) Tumor cells showing positive expression for OCT 3/4 immunohistochemical stain.

The surgically resected specimen was then sent for histopathological analysis, which revealed a neoplastic lesion in the left ovary arranged in nests and trabeculae separated by fibrous septa. The cells were polygonal with moderate amount of clear to eosinophilic cytoplasm. The nuclei were round to oval, showed moderate plemorphism with prominent eosinophilic nucleoli and frequently visible mitotic activity. Scattered plasma cells and lymphocytes were present within the fibrous septa. In areas, tumor cells were seen scattered against edematous stroma. Thin-walled dilated and congested blood vessels were seen, suggestive of vascular compromise secondary to torsion. The neoplastic cells showed diffuse nuclear positive expression for octamer-binding transcription (OCT) 3/4 immunohistochemical stain. Intracytoplasmic glycogen was highlighted on Periodic acid-Schiff special stain (Figure 4).

The overall findings confirmed mature ovarian dysgerminoma that was limited to the left ovary without capsular invasion (TNM stage: T1A, N0, M0 according to FIGO staging). The excised left fallopian tube, omentum, and peritoneal washout were all negative for malignancy.

Postoperatively, the patient showed satisfactory progress and was therefore discharged in a stable condition. She is planned for on oncology follow-up visit as an outpatient.

## Discussion

It is a well-known fact that mobile organs and pedunculated masses are prone to acute torsion, which leads to devascularization of the organ or tumor. The pathophysiology of acute torsion are initially venous and lymphatic obstruction, which result in massive intratumoral edema followed by progressive arterial compromise, which predisposes to gangrene, hemorrhagic infarction, and rupture [3]. Adnexal torsion in young females that are induced by ovarian masses are usually due to benign causes such as physiological cysts, endometriomas, and benign tumors like dermoids [4]. Very few reports of adnexal torsion due to malignant ovarian dysgerminoma have been reported in the literature, especially in the pediatric age group. According to a study by Lee et al., torsion of ovarian tumors mostly occurred in the reproductive age group, more commonly on the right side, and only approximately 8% of masses were malignant [5].

Delay in the diagnosis may occur if the torsion is partial and intermittent with subsequent spontaneous detorsion, in which case the symptoms may subside, only to return within hours, days, or weeks. We believe our patient also had intermittent torsion and detorsion leading to on and off symptoms that lasted for a week before presentation. Moreover, the condition can be clinically mistaken for acute appendicitis, diverticulitis, tubo-ovarian abscess, ectopic pregnancy, and ruptured ovarian cyst given the similar clinical presentation. Ultrasonography is usually considered the initial imaging modality of choice and can demonstrate the twisted vascular pedicle with decreased vascularity of the tumor on color Doppler. However, ultrasonography may be equivocal and inconclusive depending on the degree of torsion and the experience of the operator. In such cases, and especially when patients present with an acute abdomen as in our case, CT can help to confirm the torsion, detect complications, and exclude other pathologic conditions quickly and effectively [3].

The ovarian vascular pedicle anatomically comprises of the gonadal vessels exiting and entering the ovary. If an ovarian mass is present, the ipsilateral ovarian vessels may be enlarged. Therefore, the “ovarian vascular pedicle sign” as suggested by Lee et al. is also a useful sign to determine the exact site of origin of the mass, especially in cases of ambiguous locations due to large size of the mass or when complications like torsion arise [6]. The presence of a twisted vascular pedicle with whirlpool sign, decreased enhancement of the tumor, intratumoral hemorrhage or edema, rupture, peritumoral stranding, and free fluid with or without hemoperitoneum are findings that are strongly suggestive of torsion on CT [3]. Areas of low attenuation in the tumor, speckled calcification, ovarian vascular pedicle sign, and free pelvic and perilesional fluid on CT all stood true in our case. Another important radiological feature of ovarian dysgerminomas is the presence of vascular septa, which show marked enhancement on arterial phase on both CT and magnetic resonance (MR), as suggested by Tanaka et al. [7]. Since our patient had torsion of the tumor, these enhancing septa were not visualised in our case contrary to usual reports, which may be a distinguishing feature in cases of torsion possibly owing to vascular compromise.

The primary goal of radiological investigations includes characterization of the lesion, staging, and evaluation of possible acute complications like torsion, tumor rupture or hemorrhage, which if passed undiagnosed may significantly increase disease morbidity and mortality. According to a report by Takeda et al., unilateral salpingo-oophorectomy via laparoscopic approach has been proposed as a reasonable management strategy for stage IA disease rather than a more extensive exploratory surgery [4]. However, laparotomy has always remained a gold standard treatment strategy as compared to laparoscopic surgery for tumors greater than 10 cm in size [8]. Once diagnosed, ovarian dysgerminomas respond well to chemotherapy, potentially sparing patients from infertility and associated morbidity. Five-year survival rate for stage I disease is reported to be 96%, with five-year survival rate of more than 80% for disease recurrence or advanced disease at the time of diagnosis [9].

## Conclusions

In summary, we report a rare presentation of ovarian dysgerminoma with torsion in a young child. Even malignant ovarian germ cell tumors like dysgerminomas can present with complications like torsion, rupture or hemorrhage and should always be considered in the differential diagnosis of young females presenting with acute abdominal pain and a palpable abdominopelvic mass. As the tumor is chemosensitive, early diagnosis by distinct radiological features can result in a good prognosis and thus reduce disease morbidity and mortality to a substantial extent.
